# The ejection of large non-oscillating droplets from a hydrophobic wedge in microgravity

**DOI:** 10.1038/s41526-021-00182-4

**Published:** 2021-12-17

**Authors:** Logan J. Torres, Mark M. Weislogel

**Affiliations:** 1 IRPI LLC, Wilsonville, OR USA; 2 Portland State University, Portland, OR USA

**Keywords:** Engineering, Aerospace engineering

## Abstract

When confined within containers or conduits, drops and bubbles migrate to regions of minimum energy by the combined effects of surface tension, surface wetting, system geometry, and initial conditions. Such capillary phenomena are exploited for passive phase separation operations in micro-fluidic devices on earth and macro-fluidic devices aboard spacecraft. Our study focuses on the migration and ejection of large inertial-capillary drops confined between tilted planar hydrophobic substrates (a.k.a., wedges). In our experiments, the brief nearly weightless environment of a 2.1 s drop tower allows for the study of such capillary dominated behavior for up to 10 mL water drops with migration velocities up to 12 cm/s. We control ejection velocities as a function of drop volume, substrate tilt angle, initial confinement, and fluid properties. We then demonstrate how such geometries may be employed as passive no-moving-parts droplet generators for very large drop dynamics investigations. The method is ideal for hand-held non-oscillatory ‘droplet’ generation in low-gravity environments.

## Introduction

Liquid drop dynamics is a large field of research within the fluid mechanics discipline. In general, provided buoyancy and inertia are sufficiently low, wall-bound drops and free drops assume constant curvature minimal surface energy states attributed to capillary forces due to surface tension. For poorly or nonwetting wall-bound liquid drops in the air, the force of gravity quickly overwhelms that of surface tension as drop volumes increase. Such large drops are better described as puddles^1^, with drops better identified for small volumes *V* $\lesssim$ (*σ*/*ρg*)^3*/*2^, where *σ* is the liquid surface tension, *ρ* is the density difference across the liquid interface (≈*ρ*_l_, the density of the liquid), and *g* is the acceleration field strength, i.e., gravity with *g*_o_ = 9.8 m/s^2^. For free drops, inertial forces such as the drag from the surrounding gas of density *ρ*_g_ and characteristic velocity difference *U* can also quickly overwhelm those of surface tension such that we expect drop volumes limited to *V* $\lesssim$ 4^4^(*σ*/*ρ*_g_*U*^2^)^3^. In terrestrial environments, *U* is often dependent on *g*; i.e., for falling drops of characteristic radius *R*, *U* ~ (8*ρ*_l_*Rg*/3*ρ*_g_)^1/2^, and *V* $\lesssim$ (27/2)(*σ/ρ*_l_*Rg*)^3^; larger drops break up until the latter condition is satisfied.

Thus, in terrestrial environments, wall-bound drop volumes are approximately limited to *V* ~ *g*^−3/2^ and free drops to *V* ~ *g*^−3^. In either case, capillary oscillation and viscous settling times are characterized by *τ*_cap_ ~ (*ρ*_l_*V*/*σ*)^1/2^ and *τ*_visc_ ~ *V*^2/3^/*ν*, respectively, where *ν* is the kinematic viscosity of the liquid. From such relationships it is easy to see how significant reductions in gravity level dramatically increase liquid volumes that might remain categorized as ‘droplets.’ For example, in the nearly weightless environment of orbiting or coast spacecraft, local body force accelerations are indeed low, with *g* ~ 10^−8^*g*_o_ reported for free fliers^2^, and with ‘microgravity’ conditions common for crewed vehicles where *g* ~ 10^−6^*g*_o_. In microgravity, wall-bound drop volumes increase ~10^9^-fold, capillary response times increase ~10^4^-fold, and viscous settling times increase ~10^6^-fold. Increases in free drop properties are larger: drop volumes ~10^18^-fold, capillary response times ~10^9^-fold, and viscous times ~10^12^-fold. Because spacecraft employ large liquid inventories such as propellants, fuels, cryogens, coolants, and water, they are not only facilities aboard which to further investigate enormous drop dynamics phenomena, they are also vehicles aboard which such phenomena may arise routinely in engineering systems. One critical example is the destabilization of fluid surfaces in large ~ 8 m diameter partially filled liquid fuel tanks in response to any number of perturbations to the spacecraft such as aerodynamic drag, stage separation, docking, thrust resettling, orbital maneuvers, and others.

In this paper, we introduce and analyze a simple method for producing large oscillation-free liquid drops for subsequent investigations and applications. The method exploits the microgravity environment to form and deploy such drops, the dynamics of which may then be studied in a microgravity environment. These large drops require significant time to form and eject—up to tens of minutes aboard orbiting spacecraft^3^, minutes aboard sounding rockets^4^, and seconds in terrestrial drop tower facilities^5^.

In fact, robust large drop formation and deployment in microgravity conditions can be a challenging task. For example, the most common method involves the growth and detachment of a pinned drop at the tip of, say, a syringe needle^6^. During the growth phase, the drop often de-pins from the needle tip and wets its outside surface leading to asymmetries and deployment failure. Even when pinning is perfect the detachment process frequently leads to rivulet rupture, satellite droplet production, ingestion of small bubbles, and perturbations to the drop in the form of low-frequency and high-frequency capillary waves, uncontrolled translation, and potentially undesirable rotation. Other methods with similar challenges may be cited^7–10^.

**Fig. 1:**
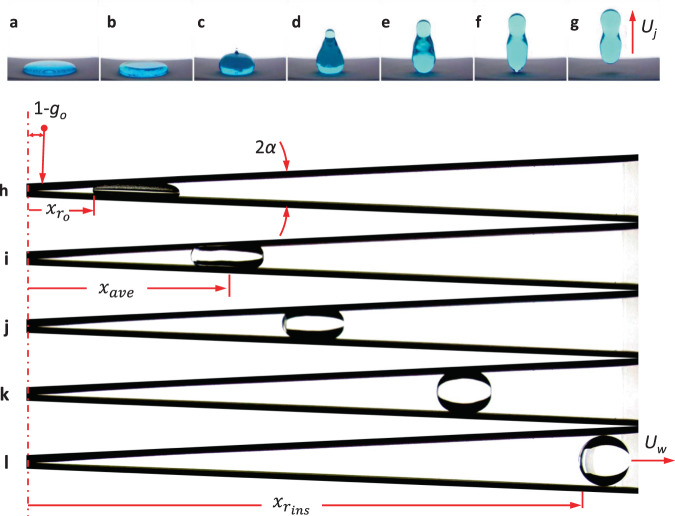
‘Low-gravity’ puddle and SH-wedge droplet ejection in drop tower tests. **a**–**g** 20 Hz sequence of 5 mL dyed water puddle jump from a textured PTFE-coated hydrophobic substrate with *θ* = 150° following the step reduction in gravity during simple drop tower test: **a** static 1−*g*_o_ interface, **b** inward radial capillary wave formation, **c**–**e** geyser formation, and **f**, **g** detachment at steady velocity *U*_j_. **h**–**l** Drop tower test of 5 mL ‘puddle jump’ in SH-wedge of *α* = 2.5°: **h** static 1−*g*_o_ puddle, **i**–**k** acceleration under internal capillary pressure-driven flow, and **l** ejection at *t* = 1.9 s with non-oscillating steady velocity *U*_*w*_ ≈ 10 cm/s.

Several recent investigations^11,12^ have demonstrated a method for large drop ejection from puddles that jump spontaneously from hydrophobic surfaces during routine 2.1 s drop tower tests—the puddle at 1−*g*_o_ becomes a drop in 0−*g*_o_. Figure 1a–g illustrates such puddle jumping for a 5 mL water puddle on a textured PTFE surface with static contact angle *θ* = 150°. Despite the short duration of the drop tower test, low-*g* drop volumes produced in this manner can be over 30,000-fold larger than similar terrestrially investigated drop phenomena^13^. Unfortunately, the sudden reduction in gravity initiates a radially inward capillary wave that constructively interferes at the drop axis resulting in an increasing variety of events with increasing puddle volume; i.e., geyser formation, satellite drop ejection, bubble ingestion, drop fission, and large amplitude underdamped oscillations with damping times far longer than the drop tower test time available (ref. Fig. 1d–f). For water puddles between 0.04 and 400 mL, drop detachment occurs within between 0.1 and 2.0 s with ejection velocities *U*_j_ between 0.7 and 12 cm/s (ref. Fig. 1g). Discrepancies and irregularities are observed for large puddles where initial condition symmetry is difficult to achieve for the nonwetting puddles in a 1−*g*_o_ environment.

In the case of puddle jumping employed as a method to deploy large drops in low-*g* environments for further research, many interesting phenomena related to that process can be studied. The behavior of recoiling non-wetting moving contact lines, inertial contact lines^14^, highly inertial nonlinear capillary surface oscillations, analogies to droplet rebound phenomena, and numerical method benchmarking are a few such studies. The puddle jumping method is particularly useful in drop tower tests due to its simple implementation. Unfortunately, the large amplitude under-damped oscillations created during the puddle jump sequence add complexity to further interactions downstream. We note that the puddle jump method is capable of such non-oscillatory drop ejections, but only for a restricted class of highly viscous liquids^15^.

However, if such a method could deploy equally large though significantly less perturbed drops, further uniquely low-*g* drop dynamics investigations could be pursued such as the study of large dynamic drop wall impact investigations, Leidenfrost drop impacts, electrostatic droplet manipulation studies^16^, and others. We pursue the present work in hopes of establishing such a method using a super-hydrophobic (SH-) wedge. We study the performance of the device from a primarily empirical perspective.

In general, we seek a passive large drop deployment method that ejects large non-oscillating drops in the brief low-*g* environment established in a 2.1 s drop tower. As shown in Fig. 1h–l, we modify the puddle jump method by adding a tilted SH plate just above the 1−*g*_o_ puddle. As the puddle begins to recoil following the step reduction of *g*-level during the drop tower test, a capillary pressure gradient is established in the partially confined liquid which drives it out of the wedge as a large drop, largely free of oscillations.

The passive migration of gas bubbles and *wetting* liquid slugs in acute wedges has been studied extensively with the wedge alternatively referred to as an interior corner, interior edge, a tapered channel, non-parallel plates, tilted plates, etc. Terrestrial research regarding micro-fluidic applications is reported by myriad authors^17–24^ with demonstrations of passive bubble removal from multi-phase flows in microgravity provided by only a few^25–27^.

Due in part to the limited access to low-*g* environments, less attention has focused on inertial-capillary dominated liquid drops in *non-wetting* wedges^28^ satisfying *θ* > *π*/2−*α*, where *θ* is the equilibrium contact angle of the surface and *α* the wedge half-angle. The short-term goal of this work is to observe how the wedge geometry controls ejection velocity for large drops while considering the limited time for ejection afforded by drop towers. We highlight design guides for large quiescent drop generators for follow-on research conducted in drop towers as well as in other reduced gravity facilities (i.e., parabolic aircraft, suborbital rockets, and spacecraft).

## Results

### Ejected drop position and velocity: experiments and predictions

**Fig. 2:**
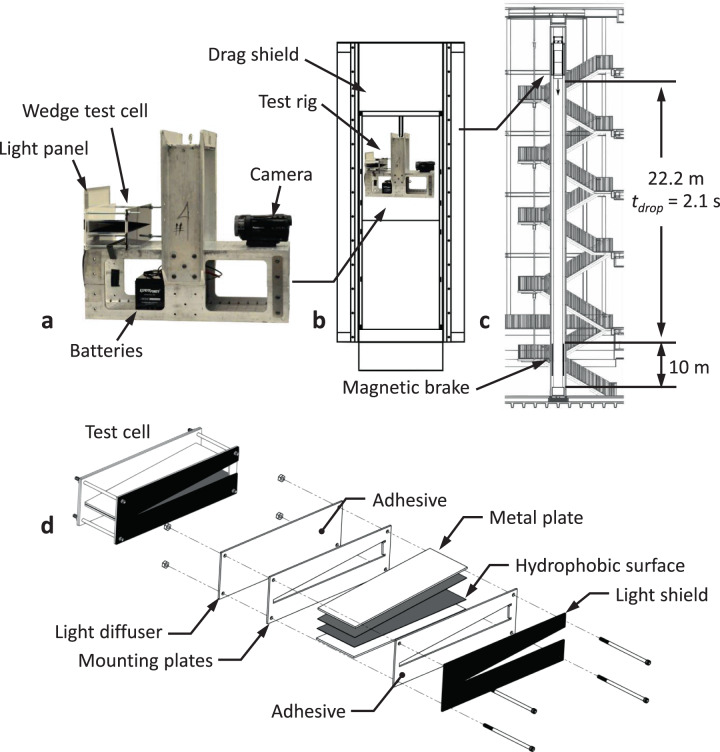
Drop rig, drop tower facility, and wedge test cell. **a** Image of experiment rig, **b** suspended within drag-shield, and **c** placed in the drop tower. **d** An exploded view of an SH-Wedge test cell of wedge half-angle *α*.

**Fig. 3:**
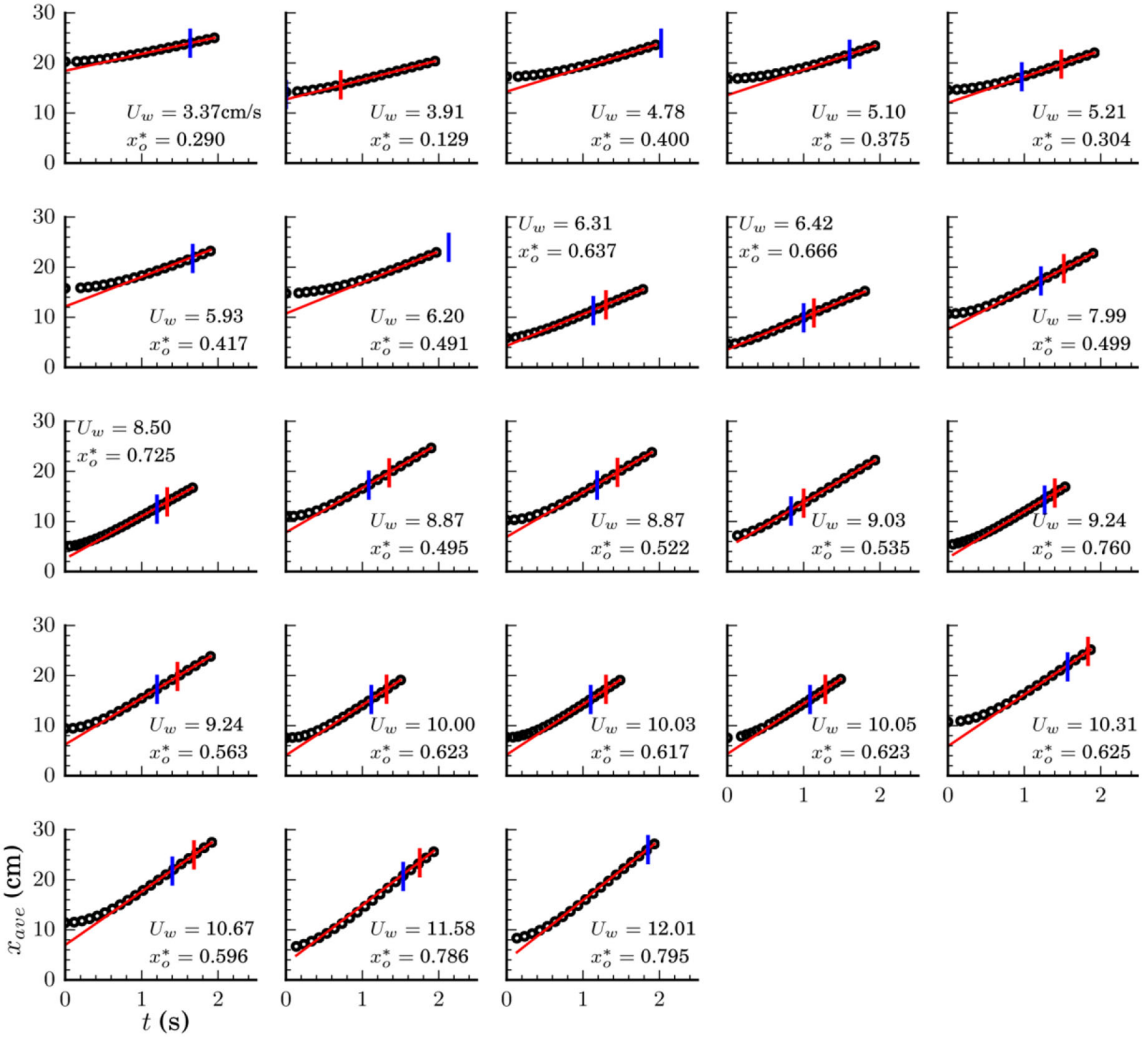
Collage of capillary migration drop tower experiments (ref. Supplementary Table 1). Transient location of averaged drop position *x*_ave_ (cm) vs. *t* (s) for wedges 1.0˚ ≤ *α* ≤ 3.8˚ and drops $0.5 \le V \le 10$ mL with varying initial locations resulting in confinements $0.1 \le x_{{o}}^ \ast \le 0.80$. Plots are arranged in order of increasing maximum drop velocity *U*_w_ which generally increases with $x_{\mathrm {o}}^ \ast$. Inscribed drop locations are noted by red tick marks when achieved. Static equilibrium locations are noted by blue tick marks.

As annotated in Fig. 1, drop tower experiments depicted in Fig. 2 are conducted to determine drop locations *x*_ave_(*t*) as functions of time for a variety of drop volumes *V*, drop initial locations $x_{r_0}$, and wedge half-angles *α*. The transient average drop locations *x*_ave_ for all experiments are presented in Fig. 3, with details provided in Supplementary Table 1. The wedge ejection velocities *U*_*w*_ are calculated from central differences and identified by linear red slopes. The red ticks indicate the time *t*_w_ at which the drops pass through their inscribed locations $x_{r_{{\mathrm {ins}}}}$. Drop trajectory histories that do not include such tick marks do not fully eject within the drop time available $t_{{w}} > t_{{{drop}}}$. Such ‘no-ejection’ drops are in the process of moving through the wedge. They achieve the constant ‘ejection’ velocity but do not pass the inscribed location $x_{r_{{\mathrm {ins}}}}$. In all tests presented, the drop behavior is characterized by a transition to constant velocity. Experiments conducted that do not exhibit a constant maximum drop velocity (linear regions in Fig. 3) are not reported.

**Fig. 4:**
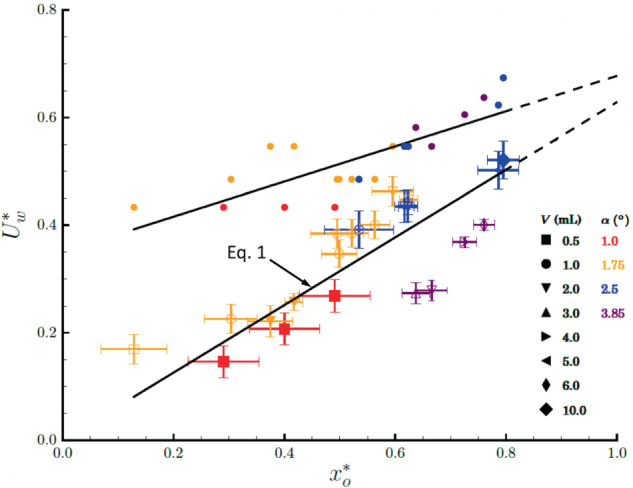
Normalized drop velocities $U_{{o}}^ \ast = U_{{w}}/\tilde U$ versus capillary confinement parameter $x_{{o}}^ \ast$. Experimental data is denoted by symbols with error bars for constant drop velocity ejections. The solid symbols identify tests that did not reach *x*_ins_, but do establish *U*_*w*_. Corresponding theoretical velocities are represented by small, closed circular symbols without error bars identified from the cylindrical puddle surface energy difference approximation of Eq. (5). An approximate linear trend in both experimental (from Eq. (1)) and theoretical velocities are observed and shown using the black overlayed lines, with both tending towards agreement as $x_{{o}}^ \ast \to 1$, shown by the extrapolated converging dashed lines.

The experimental average exit velocities are normalized by the theoretical maximum velocity $\tilde U$ (ref. Eq. (5)) as $U_{{w}}/\tilde U \equiv U_{{w}}^ \ast$ and plotted against the capillary confinement parameter $x_{{o}}^ \ast$ (ref. Eq. (7)) in Fig. 4 using solid symbols for non-ejected drops and open symbols for ejected drops. Horizontal and vertical error bars provide measurement uncertainty, with an average relative uncertainty of ±10%. Repeatability of a 2 mL drop in a wedge of half-angle $\alpha = 2.5^\circ$ at $x_{{o}} \approx x_{{{cap}}}$ for $x_{{o}}^ \ast = 0.62$ is observed to be *U*_*w*_ = 10.03 ± 0.02 cm/s for three tests conducted.

The predicted velocity (from Eq. (5)) is presented in Fig. 4 using small, closed circular symbols. The model over-predicts velocity for all $x_{{o}}^ \ast$ as expected, but with decreasing error as $x_{{o}}^ \ast \to 1$ where the model assumptions are most appropriate. An average model discrepancy of ±67% is achieved without consideration of complex effects due to transients, refined geometry, moving contact line dynamics, and others. An approximate linear trend is observed for both experimental and theoretical velocities in Fig. 4, the experimental data being correlated for wedge design purposes by

1 $$U_{{{wl}}}^ \ast = 0.64\;x_{{o}}^ \ast ,$$

providing a quick estimate with ±18% average error.

**Fig. 5:**
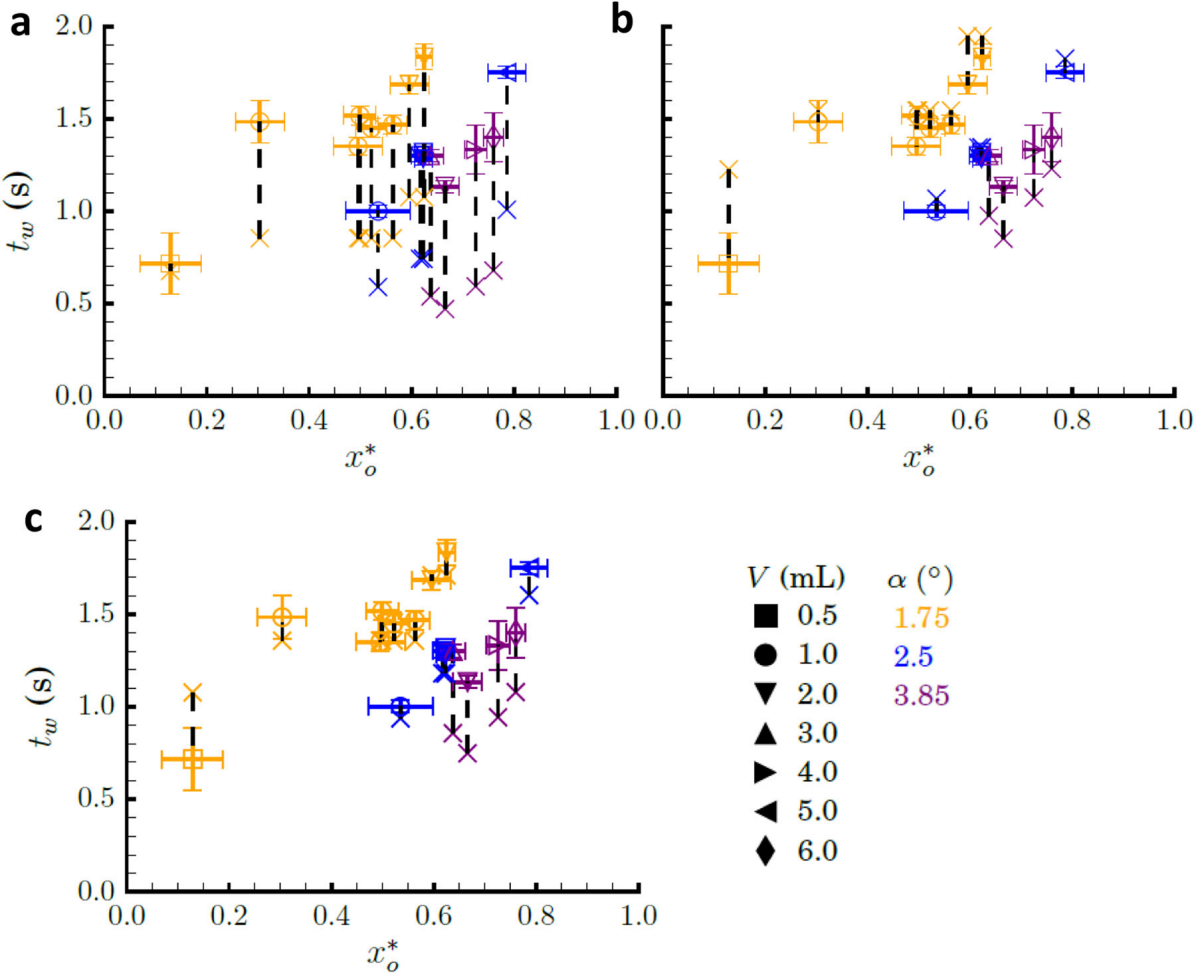
Experimental (open symbol) and theoretical (x symbol) ejection times *t*_*w*_ with respect to the capillary confinement parameter $x_o^ \ast$. The vertical dashed lines associate the theoretical prediction with the corresponding experimental measurement. Legend at lower right. **a** Eq. (6), **b** Eq. (2), and **c** Eq. (3).

Ejection times for drops that achieve *x*_rins_ within *t*_drop_ are presented in Fig. 5. Predicted ejection times *t*_w_ (from Eq. (6)) are plotted with the data in Fig. 5a. As anticipated, the predicted times are shorter than observed in the experiments. A correction of the form

2 $${{t}}_{{w}_{\,corr}} = 1.82\left( {\frac{{\rho R_{{s}}^4}}{{4\sigma g_{{o}}}}} \right)^{1/4}\left( {\csc \alpha - 1} \right),$$

yields an average error of ±14% as shown in Fig. 5b. Additionally, using the linear model ejection velocities $U_{w_l} = 0.64\;\tilde Ux_o^ \ast$ from Eq. (1) and the distances to the inscribed locations $x_{r_{ins}} - x_{r_o} \equiv {\Delta}x$, the form of Eq. 2 is re-derived from $t_{w_l} = {\Delta}x/U_{w_l}$ yielding

3 $$t_{{w}_{\,corr}} = 1.56\left( {\frac{{\rho R_{{s}}^4}}{{4\sigma g_{{o}}}}} \right)^{1/4}\left( {\csc \alpha - 1} \right),$$

with an average error of ±16%, as shown in Fig. 5c. No specific trend in ejection times are found with respect to $x_o^ \ast$. However, the form of Eq. (3) derived from the linear capillary confinement parameter ejection velocity model closely matches the corrected ejection time of Eq. (2) which is derived from first principles. Thus, either Eq. (2) or (3) can serve adequately for wedge drop generator design.

## Discussion

The hydrophobic wedge geometry provides an attractive degree of control for use as a passive, large volume, low-*g* drop generator. The nearly non-oscillating drops generated during the experimental drop tower investigation range in volumes from 0.5 ≤ *V* ≤ 10 mL with ejection velocities between $3.37 \le U_w \le 12.01$ cm/s and oscillation amplitudes typically <3% of spherical radius dimension at ejection. Without the time limitation of a drop tower, significantly larger and slower drops could be ejected for a variety of fundamental and applied investigations. For example, low-speed impacts of large non-oscillating drops on heated surfaces above the Leidenfrost temperature could be uniquely studied employing the hydrophobic wedge geometry.

The results provided suggest the use of the capillary confinement parameter $x_{\mathrm {o}}^ \ast$ and its correlations to predict wedge drop generator performance. The confinement parameter combines drop volume, drop initial condition/position, and wedge half-angle into a single term that can be used to approximate drop ejection velocity and ejection time for a hydrophobic wedge (Eqs. (1) and (3), respectively). Alternatively, the semi-empirical ejection time of Eq. (2) also provides predictions based on an ideal energy transfer velocity limit, closely matching the empirically derived ejection model time Eq. (3). For the 23 nominal drop tests presented, average prediction uncertainties in Eqs. (1)–(3) are <± 20%. With such tools in hand, one can expect to eject drops of volume *V* ~ 2 mL with ejection velocities *U*_*w*_ ~ 9 cm/s in 0.5 s drop towers, *V* ~ 10 mL at *U*_*w*_ ~ 12 cm/s in 2 s towers, and *V* ~ 30 mL at *U*_*w*_ ~ 13 cm/s in 5 s towers.

**Fig. 6:**
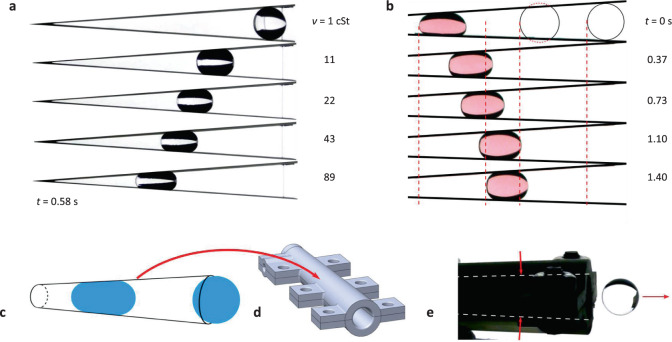
Viscous effects, contact angle hysteresis effects, and a conical SH droplet shooter. **a** Drop tower images at *t* = 0.58 s of 1 mL viscous drops ranging in kinematic viscosity from 1 to 89 cSt. Increased viscosity decreases the viscous time scale *t*_ν_ = *L*_s_^2^/*ν*, thus initiating early transition to constant velocity as well as decreased ejection velocity. **b** A time sequence of a 0.2 mL drop in hydrophobic wedge of *α* = 2.8^°^ during a drop tower test. By *t* = 1.4 s the drop has slowed and stopped by contact angle hysteresis. **c** Schematic of a conical hydrophobic drop generator design and **d** 3D-printed model constructed as two halves. **e** Drop tower test image of a 0.71 mL drop ejecting from the *α* = 2° cone with velocity *U*_*w*_ = 7.2 cm/s.

The present work serves as a foundational demonstration for continued investigations in drop migration in non-wetting tapered geometries. The energy model presented in the “Methods” section is a zeroth-order approximation, accounting for inertial and capillary energies over viscous losses and contact angle hysteresis. The drop time in all cases presented is less than the viscous time scale $t_{{\mathrm {drop}}} < t_\nu$. Future work may seek to expand the analytic model to such effects to increase accuracy as well as adapt the model for fluids of various viscosities. Additionally, identification of the no-ejection limit for wedge bound drops is of interest and expected as drop volume *V* and wedge half-angle *α* decrease. A few experiments regarding these topics were carried out. Figure 6a presents snapshots of five drop tests of 1 mL drops in an *α* = 4° wedge with varying kinematic viscosity $\nu$. As expected, increased viscosity serves to reduce ejection velocity for drops of the same initial confinement parameter $x_o^ \ast$. Figure 6b time sequence of a small volume *V* = 0.2 mL drop inside a *α* = 2.7° wedge demonstrates a no-ejection regime where the drop transitions to a final resting location $x_{{\mathrm {r}}_{\mathrm {f}}}$ set by contact angle hysteresis, in contrast to the inertial-capillary ejected drops studied throughout the preceding sections.

Other tapered hydrophobic geometries, such as a cone or rectangular diffuser, are also of interest for future investigations and applications. A tapered hydrophobic cone varies from the wedge in a few interesting ways. For instance, a capillary drop will occlude the entire cross-section of a cone, restricting air flow from the advancing side to the receding side of the drop. For migration to occur, air must flow through the vertex of the cone or around the drop along the hydrophobic surface. The drop migration velocity is thus air flow limited. This feature can be used to reduce the drop migration velocity to nearly zero. A single demonstrate of a conical drop generator is shown in Fig. 6c–e. A 3D-printed cone with *α* = 2° in Fig. 6d is shown in Fig. 6e image with a 0.71 mL drop ejecting with steady velocity 7.2 cm/s.

**Fig. 7:**
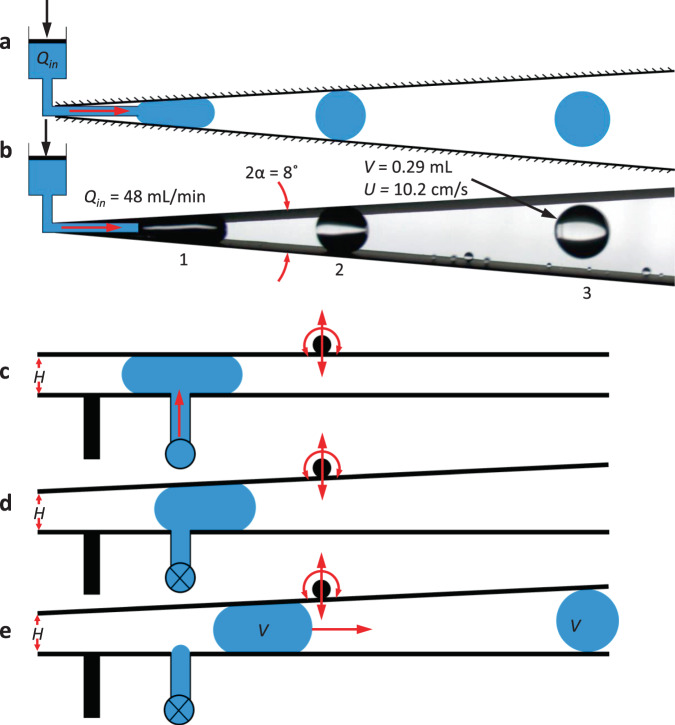
Steady periodic droplet ejection and schematic for hand-held low-g wedge drop generator. **a** Example schematic of a continuous wedge drop generator through growth and detachment of drops near the apex of a planar hydrophobic wedge. **b** Image of drop tower test demonstration where a water stream of *Q*_in_ = 48 mL/min is injected into an *α* = 4° wedge, breaking up into 0.29 mL drops with ejection velocity *U*_*w*_ = 10.2 cm/s. **c** A desired volume of liquid is injected between parallel hydrophobic surfaces, **d** the upper surface is rotated to desired wedge angle until in **e** the drop migrates and ejects at right.

Further research seeks to implement the wedge drop generator into capillary drop dynamics investigations where large quiescent slow-moving drops are required, particularly for space experiments where the duration of microgravity is essentially unlimited. Such an environment is conducive to large sample size experiments, which require continuous deployment of identical drops. Integration of a hydrophobic wedge and a fluid injection system could meet such a requirement. Figure 7a presents a simple schematic for a system using a hydrophobic wedge and syringe pump. Fluid is injected at a steady rate *Q*_in_ near the apex of the wedge where it grows into a large drop until detachment occurs and the drop migrates away under the capillary pressure. Fig. 7b provides a drop tower demonstration of Fig. 7a. A single image captures three drops of volume *V* = 0.29 ± 0.01 mL in an *α* = 4° wedge at stages of (1) growth, (2) detachment, and (3) ejection, with eventual ejection velocities of $U_{{w}} = 10.2 \pm 0.1$ cm/s for the drops from a constant water flow rate of $Q_{{\mathrm {in}}} = 48$ mL/min. Further tests could be performed to investigate the effect of wedge geometry, fluid properties, and injection rate on ejection time, ejection velocity, drop oscillation, frequency, and amplitude.

A drop-on-demand device could integrate the wedge geometry into a hand-held ‘drop shooter’ for astronaut demonstrations and experiments. Figure 7c–e schematics outline a three-step hand-controlled process. In Fig. 7c a drop of desired volume is deposited between two parallel planar hydrophobic surfaces with prescribed uniform initial separation *H*. In Fig. 7d the top surface is rotated until the downstream opening reaches the required diameter for the injected drop 2*R*_s_. In Fig. 7e the drop migrates along the device eventually ejecting at the opening. Such a device could be operated to produce a range of large oscillation-free low-*g* drop ejection volumes and velocities to accommodate the desired drop dynamics investigation requirements.

In general, large, nearly oscillation-free drops can be passively deployed in low-*g* environments by injecting liquid into a simple superhydrophobic wedge. The wedge geometry damps large oscillations of the initially distorted drops (i.e., puddles) on the way to becoming spherical drops in microgravity environment. Drops of volumes 0.5–10.0 mL ejected at velocities 3.4–12.0 cm/s were demonstrated herein during the short-duration experiments performed in a 2.1 s free fall drop tower. The impacts of initial drop confinement, wedge half-angle, viscosity, orientation with respect to gravity, and contact angle hysteresis were investigated in a cursory manner. A means of estimating ejection velocity to within ±20% is provided as a function of drop volume, initial position, and wedge half-angle for water drops. The method may be uniquely suited for further large droplet impact investigations using drop towers, parabolic aircraft, suborbital rockets, or orbiting or coast spacecraft.

## Methods

### Experiments

All our experiments are conducted using the 2.1 s Dryden Drop Tower located at Portland State University, which is depicted schematically in Fig. 2. The 22.2 m tall tower provides a brief 2.1 s free fall period with maximum acceleration $\lesssim$10^−4^*g*_*o*_. The test rig shown in Fig. 2a contains all necessary experiment components including a wedge test cell, diffuse LED backlight panel, batteries, and HD Panasonic HC-WX970 120 fps video camera. The experiment rig experiences low acceleration during its decent due to the drag shield.

A typical SH-wedge test cell is shown in exploded view in Fig. 2d. Aluminum Oxide 320-grit sandpaper adhered to a metal plate via transfer tape is spray-coated with a polytetrafluoroethylene-based (PTFE) commercial aerosol spray (King Controls Magic Dome) to create planar marginally super-hydrophobic substrates with average apparent static contact angles of *θ* = 147 ± 3°, as determined by height-width measurements of 10 low-Bond number sessile drops on each surface. For the large drop volume tested roll-off angles are indeed low <2°. The assembled wedge test cell is fixed to the test rig with the lower wedge substrate perpendicular to gravity. With the assembled test rig and test cell hanging inside the drag shield, a drop of distilled water is delicately deposited onto the lower face of the SH-wedge via calibrated graduated syringe and then delicately rolled to the desired wedge location by slightly pitching the test rig.

Images of the spontaneous low-*g* flow phenomena as shown in Fig. 1h–l are captured and analyzed via open-source image analysis software FIJI^29^. Reduced data such as wetted planar area and front, back, and centroid locations are gathered. Over 200 drop tower tests were conducted to investigate or utilize the wedge ejector. A selection of 29 of these tests that support the present work are summarized herein, specific details of which are included in Supplemental Tables 1 and 2. These experiments are discussed in the previous sections, while a brief review of the driving forces of the phenomena will follow. Sample trajectory plots from tests varying drop volume, wedge angle, and initial position are provided in Fig. 3 which show average drop position in the wedge *x*_ave_ with time *t*. It is learned that highly confined droplets (i.e., large droplets in small wedge angles and close to the wedge vertex) eject with the highest velocities which are quantitatively captured by the non-dimensional capillary confinement parameter $x_{\mathrm {o}}^ \ast$. We show that for the highest quality ejections with minimal residual droplet oscillations, confinement in the wedge should be maintained at least until the droplet achieves its inscribed configuration as shown in Fig. 1l.

### Analysis

#### Capillary driving force

**Fig. 8:**
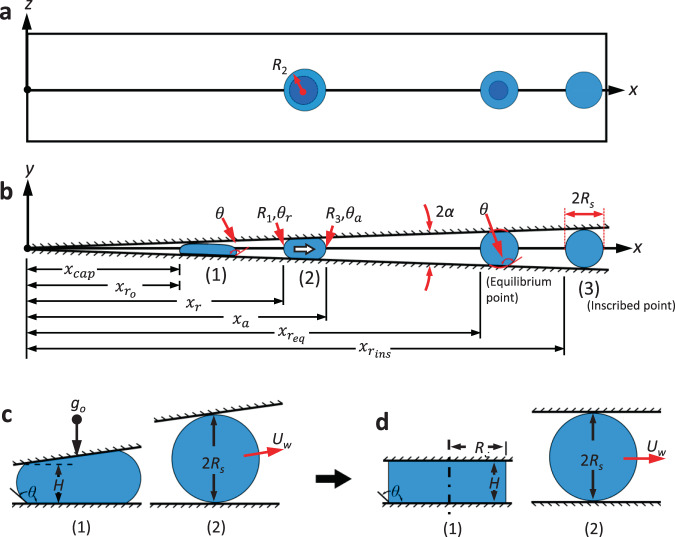
Idealized capillary drop migration in SH-wedge with idealization of initial and final puddle/drop states. **a** Top view and **b** profile view with (1) initial, (2) confined transient, and (3) inscribed states identified in **b**. Characteristic radii of curvatures *R*_1_ and *R*_3_ approximate the capillary pressure gradient inside the drop. Advancing and receding menisci represented by *x*_a_ and *x*_r_ are referenced from the wedge vertex. Initial and inscribed locations of the receding edge are identified by $x_{r_o}$ and $x_{rins}$. The approximate ‘average’ drop location is defined as $x_{{\mathrm {ave}}} = (x_{\mathrm {a}} + x_{\mathrm {r}})/2$. **c** Actual and **d** simplified models in (1) confined and (2) inscribed states. The free inscribed drop of radius *R*_s_ attains velocity *U*_w_.

The Young–Laplace equation ${\Delta}P = \sigma {{{\mathcal{H}}}}$ defines the pressure jump across a gas-liquid interface exhibiting surface tension σ and local surface curvature ${{{\mathcal{H}}}}$. Applied to the idealized capillary puddle in Fig. 8a, b, a net liquid phase driving pressure difference can be written in terms of the principal radii of curvature

4 $${{{\mathrm{{\Delta}}}}}P \approx \sigma \left( {\frac{1}{{R_3}} - \frac{1}{{R_1}}} \right),$$

where *R*_1_ and *R*_3_ are the receding and advancing radii of curvature, respectively. The radius *R*_2_ is shared by both advancing and receding curvatures and cancels from Eq. (4). The approximation of Eq. (4) applies along the positive *x*-axis for uniform planar wedges and symmetric fluid bodies in the *x–y* plane. It is this negative pressure gradient that leads to bulk motion away from the wedge vertex. Under low-*g* conditions, the gradient diminishes with $R_1 \to R_3$ as the drop acquires an increasingly spherical configuration. Concus and Finn proved the existence of a stable equilibrium point for static drops exhibiting static contact angle $\theta\, > \,\pi /2 + \alpha$, where the equilibrium radius $R_1 = R_3 \equiv R_{{\mathrm {eq}}}$ is a function of wedge geometry and wetting conditions^30^, $R_{{\mathrm {eq}}} = f(\alpha ,\theta ,V)$ (refer to Fig. 8b). However, for sufficiently large *θ*, despite viscous resistance and finite dynamic contact angle hysteresis, migrating inertial-capillary drops routinely overshoot the confined equilibrium location $x_{{\mathrm {r}}_{{\mathrm {eq}}}}$ and achieve spherical states as they pass through the inscribed location $x_{{\mathrm {rins}}} = R_{\mathrm {s}}$ (csc *α*–1) where the drop is tangent to both wedge surfaces and *R*_s_ is the spherical drop radius. Thus, for the dynamic drops of this study, Eq. (4) applies when $x_{\mathrm {r}} < x_{{\mathrm {r}}_{{\mathrm {ins}}}}$.

#### Simplified energy model

In a similar manner to Attari et al. and numerous others, a simplified energy model requires minimal analytical effort to gather insight relating to drop ejection transients and ejection velocity limits. Referring to Fig. 8c, d, the surface energy (SE) difference from confined state (1) to free state (2) is converted to kinetic energy (KE) of the bulk fluid via KE_2_ = SE_1_−SE_2_, ignoring work and dissipation during the transition. In the limit of small wedge half-angle *α* and large volumes *V*, the non-axisymmetric interface configuration of Fig. 8c (1) is approximated by the axisymmetric cylindrical disc configuration of Fig. 8d (1). Deriving the surface energies for the simplified states and solving for the bulk velocity, a modified version of the jump velocity is found to be

5 $$U_{{w}} = \tilde U\left[ { - \cos \theta + \left( {\frac{{\pi H^3}}{V}} \right)^{1/2} - \frac{{6^{2/3}}}{2}\left( {\frac{{\pi H^3}}{V}} \right)^{1/3}} \right]^{1/2},$$

where $H \equiv 2\left( {\sigma /\rho g_{\mathrm {o}}} \right)^{1/2}$ is the capillary height and $\tilde U \equiv \left( {4\sigma g_{\mathrm {o}}/\rho } \right)^{1/4}$ is the maximum theoretical velocity of the drop in the large puddle limit $\left( {\pi H^3/V} \right)^{1/3} \ll 1$ for *θ* ≈ 180°. Equation (5) resembles that derived in Attari et al., except for the contact angle term which accounts for reduced energy due to the presence of the upper surface. Dividing the maximum distance the fluid must travel to reach its inscribed length $x_{{\mathrm {rins}}}$ by the maximum ejection velocity $\tilde U$ provides a characteristic ejection time

6 $$t_{{w}} = \left( {\frac{{\rho R_{\mathrm {s}}^4}}{{4\sigma g_{\mathrm {o}}}}} \right)^{1/4}\left( {\csc \alpha - 1} \right),$$

where again $R_{\mathrm {s}} = \left( {3V/4\pi } \right)^{1/3}$. The simplified forms of Eqs. (5) and (6) serve as design guides for wedge drop generators where a specified ejection velocity is desired and where the duration of free fall is limited. The experimental validation of such predictions and their underlying assumptions are investigated in part herein.

#### Impact of initial conditions

**Fig. 9:**
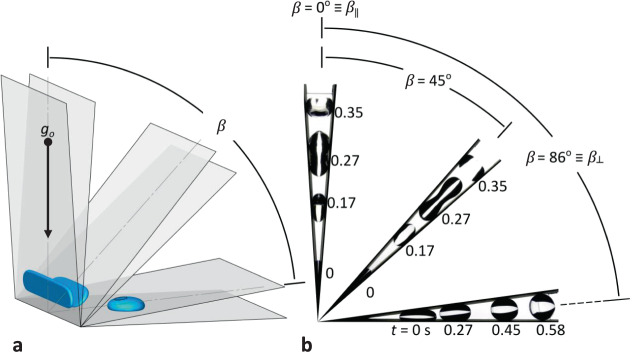
Effect of wedge angle *β* relative to gravity on oscillation amplitude and frequency at ejection location. **a** Numerically computed initial shapes of 1 mL drops in hydrophobic wedges of *θ* = 150° for *α* = 4° and *β* = 0°, 45°, and 86°, and **b** drop tower experiments of cases in (**a**).

For the drop tower experiments, a puddle of known volume is deposited at some initial position $x_{r_{\mathrm {o}}}\, < \,x_{r_{{\mathrm {ins}}}}$ within a superhydrophobic wedge. A fixed drop position is assured when Eq. (4) is either zero or balanced by another force (i.e., tilt with respect to gravity). The initial conditions are met by varying the wedge center-line angle relative to gravity as shown in Fig. 9a, the former case occurring at *β* = 90°−*α* ≡ *β*_⊥_ and the latter over the range $0^\circ \equiv \beta _\parallel \le \beta\, < \,\beta _ \bot$. When $\beta = \beta _ \bot$, gravity is perpendicular to the lower face of the wedge allowing the large drop to establish a symmetric disk-like puddle of capillary height $H \approx 2\left( {\sigma /\rho g_{\mathrm {o}}} \right)^{1/2}$ when $V \gg \left( {\sigma /{{{\mathrm{{\Delta}}}}}\rho g_o} \right)^{3/2}$. For $\beta _\parallel \le \beta\, < \,\beta _ \bot$, the drop is drawn into the vertex to various degrees by its own weight resisted only by the capillary pressure gradient Eq. (4). The drop elongates along the wedge vertex reaching a maximum length when $\beta = \beta _\parallel$. Following the effective step-reduction in the gravity of the drop tower tests, reorientation of the $\beta = \beta _ \bot$ initial condition follows that of puddle jumping (ref. Fig. 1a). However, the upper surface of the wedge suppresses the rim roll-up motion which in turn suppresses geyser formation by interfering with vertical elongation, ultimately producing a highly damped drop migration and ejection along the wedge midplane or centerline. In contrast, the minimally damped drop of $\beta\, < \,\beta _ \bot$ rolls-up along the wedge axis, forming a geyser and resulting in high amplitude deformations and under-damped oscillations during migration and ejection, characteristics similar to puddle jumping. Numerically computed initial equilibrium conditions for three configurations of *β* for a 1 mL drop in a wedge with *α* = 4° and *θ* = 150° are shown in Fig. 9a. K. Brakke’s *Surface Evolver* algorithm^31^ is implemented using the SE-FIT software^32^. Corresponding drop tower experiments are provided in Fig. 9b. The differences in drop distortion are best observed at *t* = 0.27 s with the horizontal case *β* = 86° producing the least distortion.

**Fig. 10:**
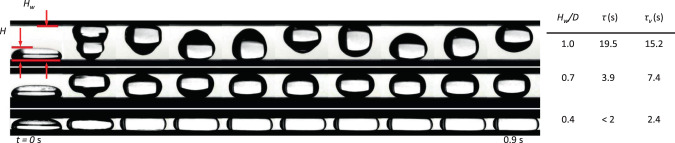
Low-g droplet rebound between parallel SH plates. 10 Hz images from drop tower tests of 2 mL water drops between parallel hydrophobic surfaces with varying separation height *H*_w_. The computed time constant $\tau = 3.91/\zeta \omega _{\mathrm {n}}$ for planar area oscillation decay decreases with the separation height *H*_w_ as predicted by the viscous time scale $\tau _\nu \sim H_{\mathrm {w}}^2/4\nu$.

The damping mechanism of $\beta = \beta _ \bot$ wedges is primarily attributed to the upper confining wall which at small *α* limits vertical drop motion during the roll-up sequence increasing interference and viscous dissipation of capillary waves during the early stages of the flow. Maximum interference and dissipation for a drop in a superhydrophobic wedge occurs as $\alpha \to 0$ with $H_{{w}} \lesssim H$, where *H*_w_ is the distance between the two surfaces. As demonstrated in the drop tower tests of Fig. 10, a 2 mL puddle rebounds between parallel hydrophobic substrates at various separation distances $H \lesssim H_{\mathrm {w}} \lesssim D$, where *D* is the drop spherical diameter. By digitally tracking the planar drop area, the characteristic time constant is $\tau = 3.91/\zeta \omega _{\mathrm {n}}$, where $\zeta$ is the damping ratio and $\omega _{\mathrm {n}}$ is the natural frequency for each height *H*_w_, and is calculated from the logarithmic decrement of successive peaks from the resultant exponentially decaying sinusoidal response. The time constant *τ* decreases with *H*_w_/*D* as listed in Fig. 10 and as predicted under the viscous time scale $\tau _\nu \sim H_{{w}}^2/4\nu$. Experiments are primarily conducted at center-line angles $\beta = \beta _ \bot$ since this initial condition produces the most uniformly damped ejected drops at $x_{{\mathrm {r}}_{{\mathrm {ins}}}}$. The receding edge initial location for drops in such wedges is bounded by $x_{{\mathrm {cap}}} \lesssim x_{{\mathrm {r}}_{\mathrm {o}}} \lesssim x_{{\mathrm {r}}_{{\mathrm {ins}}}}$, where $x_{{\mathrm {cap}}} = H/\cos \alpha \tan 2\alpha$ approximates the receding edge location where the drop just makes contact with the upper surface. Normalizing each term in this boundary inequality by $x_{{\mathrm {r}}_{{\mathrm {ins}}}}$ and subtracting each from unity produces

7 $$x_{{o}}^ \ast = 1 - x_{{o}}\left( {\frac{{4\pi }}{{3V}}} \right)^{1/3}\frac{{\sin \alpha }}{{1 - \sin \alpha }}$$

and

8 $$x_{{{max}}}^ \ast \approx 1 - 1.61\;{{{\mathrm{Bo}}}}_{{V}}^{ - 1/2} = 1 - 3.22\;{{{\mathrm{Bo}}}}_{{V}}^{ - 1/2}\frac{{\tan \alpha }}{{\tan 2\alpha (1 - \sin \alpha )}}$$

such that $0 \,\lesssim\, x_{\mathrm {o}}^ \ast\, \lesssim \,x_{{\mathrm {max}}}^ \ast$. We note that for $\pi H^3/V \equiv H^2/R_{\mathrm {c}}^2 = 1$, a zero-velocity ejection occurs at *θ* = 130° according to Eq. (5). Similarly, for $\pi H^3/V \ll 1$, we find *θ* → 90° for *U*_w_ = 0. Equation (7) provides a measure of confinement for drops satisfying $\pi H^3/V\, < \,1$. For the drop tower experiments, maximum confinement occurs as $x_{\mathrm {o}} \to x_{{\mathrm {cap}}}$ resulting in $x_{\mathrm {o}}^ \ast \to x_{{\mathrm {max}}}^ \ast$. In the limits of small wedge half-angles $\alpha \to 0$ and large drop volumes $V \to \infty$, total confinement is given as $x_{\mathrm {o}}^ \ast \to x_{{\mathrm {max}}}^ \ast \to 1$. In contrast, as $x_{\mathrm {o}}^ \ast \to 0$ no confinement is achieved. Equation (7) combines variables *V*, *α*, and *x*_o_ into a single parameter that is employed to characterize the dependent variables of the superhydrophobic wedge ejection time and velocity.

### Reporting summary

Further information on research design is available in the Nature Research Reporting Summary linked to this article.

## Supplementary information

Supplementary Information

Reporting Summary

## Data Availability

The authors declare that all data supporting the findings of this study are available within the paper.
