# Supplementary material for: The ejection of large non-oscillating droplets from a hydrophobic wedge in microgravity
Source: NPJ Microgravity. 2021 Dec 17;7:52. doi: 10.1038/s41526-021-00182-4 (PMC8683412; doi:10.1038/s41526-021-00182-4)
Supplement: Supplementary file 1 — Supplementary Information [file 41526_2021_182_MOESM1_ESM.pdf]

Supplementary Data Tables

Supplementary Table 1: Range of variables, parameters, and responses from nominal wedge experiments.

	2α °± 5%	β °± 5%	V mL ± 2%	x_{ro} cm ± 5%	x_o^* ± 10%	U_w cm/s ± 10%	t_w s ± 10%
**No. 1**	2.0	89.0	0.5	14.75	0.491	6.20	NA
**2**	2.0	89.0	0.5	17.28	0.400	4.78	NA
**3**	2.0	89.0	0.5	20.28	0.290	3.37	NA
**4**	3.5	88.3	0.5	14.20	0.129	3.91	0.88
**5**	3.5	88.3	1.0	9.42	0.522	8.87	1.52
**6**	3.5	88.3	1.0	9.45	0.563	9.24	1.53
**7**	3.5	88.3	1.0	10.69	0.499	7.99	1.60
**8**	3.5	88.3	1.0	10.96	0.495	8.87	1.43
**9**	3.5	88.3	1.0	14.59	0.304	5.21	1.60
**10**	3.5	88.3	2.0	10.75	0.625	10.31	1.94
**11**	3.5	88.3	2.0	11.42	0.596	10.67	1.75
**12**	3.5	88.3	2.0	15.78	0.417	5.93	NA
**13**	3.5	88.3	2.0	16.84	0.375	5.10	NA
**14**	5.0	87.5	1.0	7.17	0.535	9.03	1.07
**15**	5.0	87.5	2.0	7.56	0.623	10.00	1.38
**16**	5.0	87.5	2.0	7.57	0.623	10.05	1.35
**17**	5.0	87.5	2.0	7.68	0.617	10.03	1.37
**18**	5.0	87.5	5.0	6.70	0.786	11.58	1.84
**19**	5.0	87.5	10.0	8.35	0.795	12.01	NA
**20**	7.7	86.2	2.0	4.68	0.666	6.42	1.25
**21**	7.7	86.2	3.0	5.89	0.637	6.31	1.17
**22**	7.7	86.2	4.0	5.05	0.725	8.50	1.47
**23**	7.7	86.2	6.0	5.56	0.760	9.24	1.53

Supplementary Table 2: Range of variables used for a. parallel wedge and b. initial condition experiments.

**a**	2α °± 5%	V mL ± 2%	H_w cm ± 5%	H_w/D_s ± 10%	x_o^* ± 25%	**b**	2α °± 5%	β °± 5%	V mL ± 2%
**No. 1**	0.0	1.0	0.62	1.0	19.5		8.0	86.0	1.0
**2**	0.0	1.0	1.09	0.7	3.9		8.0	45.0	1.0
**3**	0.0	1.0	1.56	0.4	< 2		8.0	90.0	1.0
